# Preliminary Screening of Mineral-Based Active Packaging Films for Banana Postharvest Quality: Origin-Dependent Efficacy and Superiority of Tourmaline-Based Formulations

**DOI:** 10.3390/foods15111989

**Published:** 2026-06-03

**Authors:** Sungmo Ahn, Seokwon Lim

**Affiliations:** Department of Food Science & Biotechnology, Gachon University, Sujeong-gu, Seongnam-si 13120, Republic of Korea; dkstjdah58@gachon.ac.kr

**Keywords:** active packaging, tourmaline, zeolite, banana ripening, postharvest quality, pulp-to-peel ratio, browning index

## Abstract

Six active packaging films were prepared by melt-compounding Ti/boehmite, Zn/zeolite, and tourmaline into low-density polyethylene via masterbatch (15 wt% loading). Postharvest quality of Ecuador-, Jeju-, and Mexico-origin bananas was evaluated under sealed and perforated conditions using browning index, pulp-to-peel (P/P) ratio, and Δ°Brix. Despite high filler content, films retained adequate mechanical integrity (tensile strength 27.7 MPa; elongation 244%). Under sealed storage, zeolite-blended formulations consistently showed the lowest browning: Tour+ZL recorded 3.48% (Ecuador, day 13) and 2.99% (Jeju, day 15); T/BM+ZL recorded 5.12% and 5.22%, respectively. The single-component T/BM film showed browning comparable to or exceeding the control. Tour+ZL also maintained the lowest terminal P/P ratio for Jeju bananas (28.99%) with no decrease throughout storage, indicating superior peel moisture retention. For Mexico-origin bananas, all films failed to retard browning after day 8 regardless of composition, demonstrating that packaging efficacy is strongly origin-dependent and must be matched to commodity postharvest history rather than applied universally. Perforated packaging extended the monitorable shelf life by 6–8 days but diminished inter-film differences. Tour+ZL was identified as the lead candidate for controlled validation trials, and a cross-validated framework combining browning index with P/P ratio is proposed to detect overripening.

## 1. Introduction

Banana (*Musa* spp.) is among the most widely cultivated tropical fruits, with global production exceeding 100 million tons annually across more than 150 countries. In South Korea, banana imports reached 360,800 tons, constituting the majority of tropical fruit imports in 2025 [1]. As a climacteric fruit, banana undergoes postharvest ripening driven by endogenous ethylene, accompanied by peel browning, flesh softening, and sugar accumulation [2]. Because the bulk of this ripening occurs during the international transport, distribution, and retail-display windows that follow harvest, the development of packaging interventions capable of delaying climacteric progression under realistic logistic conditions remains a central priority for tropical-fruit supply chains [3,4]. Within this priority, the candidate-formulation landscape has expanded substantially over the past decade, encompassing chemistries as diverse as 1-MCP, photocatalytic oxides, metal–organic frameworks, carbon quantum dots, and mineral ethylene scavengers; consequently, comparative evaluation of multiple promising chemistries against bananas of differing origin and handling history has emerged as a necessary step for translating this material’s diversity into deployable technology [5,6].

A defining feature of the global banana supply chain is that fruit reaching the same retail outlet may originate from production regions with markedly different cultivar genetics, harvest maturity standards, and cold-chain durations [7]. These upstream differences modulate climacteric ethylene production, endogenous antioxidant reserves, and the activity of downstream enzymes such as polyphenol oxidase (PPO), the principal driver of peel browning [8]. As a result, packaging chemistries that perform well for one origin may underperform—or fail outright—for another, and an active packaging system that has been validated only against a single cultivar provides limited assurance of performance across the heterogeneous mixture of fruit that commercial logistics actually encounter [9]. Demonstrating both where a packaging chemistry succeeds and for which commodity profiles it ceases to be effective is therefore central to defining the operational envelope within which the technology can be reliably deployed. Multi-origin evaluation studies, conducted under the sourcing conditions actually encountered in retail logistics, are an indispensable component of this characterization, and the present work was designed around this principle [10,11].

A range of active packaging technologies has been developed to delay climacteric ripening. Classical approaches such as 1-methylcyclopropene (1-MCP) treatment, modified atmosphere packaging (MAP), and potassium permanganate sachets require auxiliary consumables or controlled application conditions, limiting their scalability in commercial logistics [2,12,13,14]. Recent advances have focused on incorporating functional materials directly into packaging films. Metal–organic frameworks (MOFs) offer tunable porosity and selective ethylene adsorption; in particular, Mg-formate MOF embedded in LDPE has been shown to delay banana ripening under simulated shipping conditions [15,16]. Carbon quantum dots (CQDs) have emerged as multifunctional additives combining UV-blocking, antimicrobial, and antioxidant activities in edible films and coatings, with recent reviews emphasizing their potential role in fresh-produce preservation [17,18]. Photocatalytic systems based on ${TiO}_{2}$ degrade ethylene via reactive oxygen species under UV or visible-light excitation, with demonstrated efficacy in tomato and banana packaging trials [5,19]. Despite the rapid pace of these advances, each technology class imposes distinct trade-offs in cost, food-contact safety clearance, processing compatibility, and operational performance under the humid, low-light conditions typical of retail banana storage; no single platform has yet been systematically validated across bananas of divergent origin within a single experimental framework.

Among these options, mineral-based fillers compounded into commodity polyolefins via masterbatch routes are particularly well-aligned with the operational realities of high-throughput banana logistics. Low-density polyethylene (LDPE) remains the dominant matrix for tropical-fruit crate-liner applications because it combines low cost, established food-contact regulatory clearance, mechanical robustness sufficient for industrial handling, and full compatibility with the blown-film extrusion infrastructure already deployed across the global produce-packaging industry [20]. Building on this matrix, recent reviews have highlighted clay- and zeolite-based mineral scavengers as leading candidates for sustainable active packaging due to their abundance, low cost, thermal stability under melt-processing, and food-contact compatibility. Within this class, the three minerals adopted in the present study were deliberately selected to span three functional categories that are individually well-documented but rarely combined within a single film: a microporous adsorbent for direct ethylene capture (zeolite) [21], a transition-metal-loaded porous oxide for catalytic ethylene removal (Ti^4+^/boehmite) [22], and a polar-crystal mineral for indirect physiological modulation (tourmaline). To date, these complementary functionalities have not been integrated within a unified LDPE-based film system designed for tropical-fruit logistics.

The rationale for combining these three minerals in a single LDPE matrix lies in the complementary nature of their mechanisms against the climacteric ripening cascade. Zeolite acts through direct physisorption of ethylene within its microporous aluminosilicate framework, whose molecular-sieve-scale pores accommodate the kinetic diameter of ethylene (≈4.2 Å), while distributed cation-exchange sites enhance uptake at the trace concentrations characteristic of fruit storage [23]. Ti^4+^-loaded boehmite (γ-AlOOH) is mechanistically distinct: rather than confining ethylene physically, its Lewis acid sites promote partial oxidation of ethylene and related volatiles, supplementing adsorption with a redox-based removal route [24]. Tourmaline, a polar borosilicate (general formula XY_3_Z_6_Si_6_O_18_(BO_3_)_3_(OH,F)_3_) with spontaneous electrical polarization, contributes through neither adsorption nor catalysis but indirectly: it emits far-infrared radiation (6–20 μm) and releases negative oxygen ions, both reported to modulate surface microbial proliferation and metabolic activity in adjacent produce [25,26]. Because physisorption, catalysis, and radiative/ionic modulation rely on fundamentally different principles, the three components act on distinct active sites and target distinct features of the postharvest microenvironment—addressing ethylene capture, volatile oxidation, and surface biological activity in parallel rather than competing for shared functional capacity. This functional complementarity, more than the performance of any single mineral, constitutes the theoretical basis for the combined formulation.

The present study addresses this gap by preparing six active packaging films incorporating finely milled porous mineral fillers into LDPE via masterbatch compounding at a total mineral loading of 15 wt%. Ethylene-scavenging mineral fillers such as zeolite are typically incorporated into fresh-produce polyolefin films at loadings of up to approximately 10 wt%, at which meaningful reductions in headspace ethylene have been demonstrated in LDPE-based systems [23,27]. Progressively higher inorganic filler contents are well-documented to degrade mechanical performance through particle agglomeration, void formation, and stress concentration [28]. The loading adopted here was therefore chosen to sit modestly above the demonstrated effective range for ethylene capture, while remaining low enough to preserve film integrity for industrial handling. Within this constraint, an asymmetric partition was used, with zeolite as the primary ethylene-adsorbing component paired with a secondary functional mineral (tourmaline or Ti^4+^/boehmite) added in smaller proportion. This arrangement preserved the dominant ethylene-capture function while allowing the secondary mineral to contribute through its own complementary mechanism. The mechanical adequacy of the resulting films at this loading is confirmed in Section 3.1. The films were applied to bananas from three commercial origins—Ecuador, Jeju, and Mexico—under sealed and perforated storage conditions. The study had three integrated objectives: (i) to identify which mineral combinations most effectively retard postharvest quality loss across multiple commodity sources, (ii) to characterize how banana origin modulates packaging efficacy as a first-order test of technology–commodity matching, and (iii) to develop a cross-validated diagnostic framework that distinguishes genuine ripening retardation from overripening-induced tissue collapse—a distinction that single-indicator (browning-only) evaluations are inherently unable to make [9,29]. Browning index served as the primary indicator, change in sugar content (Δ°Brix) as a secondary ripening-rate indicator, and the pulp-to-peel (P/P) ratio as a complementary marker for peel moisture retention and overripening detection. Together, these elements constitute a comparative evaluation framework intended to clarify both the formulations and the commodity contexts in which mineral-based active packaging delivers reliable performance.

## 2. Materials and Methods

### 2.1. Synthesis of Functional Mineral Powders

The porous carriers boehmite, zeolite, tourmaline ore and low-density polyethylene (LDPE) pellets were provided by Hangreentech Co., Ltd. (Seoul, Republic of Korea). Titanium oxychloride, zinc chloride, ammonium hydroxide, and sulfuric acid were of analytical reagent grade and purchased from Sigma-Aldrich (St. Louis, MO, USA).

Boehmite (γ-AlOOH; D_50_ = 2.33 μm; D_max = 10.35 μm; BET surface area = 312.4 m^2^/g) and zeolite (D_50_ = 3.01 μm; D_max = 8.55 μm; 285.6 m^2^/g) served as porous carriers for gas adsorption. Tourmaline ore was reduced to a film-compatible particle size distribution using a planetary ball mill (Retsch GmbH, Haan, Germany) at ambient temperature and used as-received without chemical modification.

Transition-metal loading was performed by a sol–gel impregnation method. Each carrier was dispersed in deionized water at 20% (*w*/*v*) and the slurry was stirred continuously during the dropwise addition of 0.5 M TiOCl_2_ or ZnCl_2_ using a heating mantle. The pH was maintained at 7.5–8.0 by simultaneous dropwise addition of 1 M NH_4_OH and 1 M H_2_SO_4_ with continuous monitoring using a calibrated pH meter (Orion 3-Star Plus, Thermo Scientific, Waltham, MA, USA). After impregnation at 105 °C for 24 h, the slurry was vacuum-filtered and washed at least three times with deionized water and absolute ethanol to remove residual ions, then dried overnight at 80 °C in a forced-convection oven (Daihan Scientific, Wonju, Republic of Korea) before calcination at 600 °C for 3 h in a muffle furnace under static air (Daihan Scientific, Wonju, Republic of Korea). Prior to film compounding, the untreated zeolite used as the high-loading adsorbent component was calcined separately at 500 °C for 1 h to remove residual organics and adsorbed moisture, then milled to a mean particle size below 4 μm by ball milling.

### 2.2. Film Preparation

Functional powders were compounded with LDPE resin into masterbatch (M/B) pellets as follows. Prior to compounding, the functional powders were pre-mixed with LDPE pellets at the target loading using a high-speed powder mixer (Hankook E.M Ltd., Pyeongtaek, Republic of Korea) for 10 min at room temperature. An oxidized polyethylene wax (OPE wax), a low-molecular-weight oxidized polyethylene possessing polar carboxyl and hydroxyl functional groups, was added as a dispersing agent at approximately 2 wt% relative to the masterbatch (corresponding to 0.3 wt% in the final film) to prevent agglomeration of the mineral filler particles during melt compounding. The pre-mixed batch was fed gravimetrically into a co-rotating twin-screw extruder (Hankook E.M Ltd., Pyeongtaek, Republic of Korea) with a barrel temperature profile maintained at approximately 180 °C across all heating zones; the extrudate was water-cooled and pelletized to yield masterbatch pellets of 2.5–3.0 mm in diameter. The masterbatch was subsequently blended with additional LDPE in a single-screw blown-film line to deliver the final mineral loading of 15 wt% and a film thickness of 0.05 ± 0.005 mm, which was verified at five positions across each film using a digital thickness gauge (Mitutoyo Corp., Kawasaki, Japan). The films were produced in-house at Hangreentech Co., Ltd. (Seoul, Republic of Korea). The compositions of the seven films evaluated are given in Table 1. The appearance of the seven films is shown in Figure S1A.

### 2.3. Mechanical Characterization

Tensile strength, elongation at break, and tear strength of the active films were measured at the Korea Polymer Testing & Research Institute (KOPTRI, Seoul, Republic of Korea), an ISO/IEC 17025 [30]-accredited facility, and compared with those of a conventional PE film. Tensile strength and elongation at break were determined on rectangular specimens (ca. 15 mm × 150 mm) using a universal testing machine operated at a crosshead speed of 500 mm/min at 23 °C and 50% relative humidity, following ASTM D882-18 [31,32]. Tear strength was measured by the Elmendorf method (ASTM D1922) [33] in both machine and transverse directions. For each property, at least five specimens per film were tested and the mean values are reported.

### 2.4. Banana Storage Trials

Commercially available bananas of three origins were purchased from a domestic retailer in Seoul, Republic of Korea: Ecuador (Cavendish type, imported), Jeju (Republic of Korea, domestically cultivated subtropical), and Mexico (imported). Bananas were washed with distilled water and visually graded for uniform size and color within each bunch. Because all measurements were destructive (peel separation for weighing; flesh sampling for sugar analysis), a different banana unit was used at each time point. Each packaging treatment at each time point was conducted in triplicate (*n* = 3), and significant differences among films were assessed by Duncan’s multiple range test (*p* < 0.05).

The three origins differed in visible maturity at the point of purchase: the imported fruit (Ecuador and Mexico) was predominantly yellow (approximately color index 3–4 on the Von Loesecke scale), whereas the domestically grown Jeju fruit was markedly green (approximately color index 1–2), as documented at day 0 in Figure S3. Because the fruit was obtained through commercial retail channels, the postharvest handling history of each lot was not documented and could not be independently verified. This difference in retail maturity is itself a consequence of the divergent postharvest pathways of the three origins and was not controllable under retail procurement. Fingers were detached from the crown in bunches of approximately equal size (10–12 cm in length; 120–140 g per finger). Initial quality screening excluded fruit bearing visible bruising, latex spots, or mechanical damage. After washing with distilled water and air-drying, each bunch was weighed on an analytical balance before packaging. The storage trials were conducted in a darkened laboratory environment. The ambient temperature was maintained within a range of 15–25 °C and was monitored throughout the storage period to ensure consistent conditions.

Phase 1 (sealed storage). One bunch per film was sealed in a standardized pouch (25 × 19 cm) and stored under ambient conditions (15–25 °C, dark). Representative images of sealed banana pouches at the start of storage are presented in Figure S2. Sampling intervals were Ecuador—days 0, 2, 5, 8, 11, 13; Jeju—days 0, 3, 6, 9, 12, 15; Mexico—days 0, 2, 5, 8, 11, 14. The different schedules reflect origin-specific ripening rates established in preliminary trials.

Phase 2 (perforated storage). For the two origins (Ecuador, Jeju) in which film effects were detected under sealed conditions, four perforations (ø ≈ 2 mm) were introduced into each pouch to allow limited gas exchange (Figure S1B). Measurements were taken at days 0, 3, 6, 9, 12, 15, 18, and 21. Mexico-origin bananas were excluded from Phase 2 as no packaging effect was observed in Phase 1.

### 2.5. Quality Indicators

Browning index. Standardized RGB photographs of each banana bunch were acquired against a uniform white background under fixed D65 daylight illumination using a digital camera, with fixed focal length, ISO, and white-balance settings across all time points to ensure colorimetric consistency. Images were imported into ImageJ v1.53e (U.S. National Institutes of Health, MD, USA) and converted to the HSV color space; brown regions—defined as pixels with H ∈ [0°, 50°], S ≥ 0.25, and V ≤ 0.60 following preliminary calibration against manually annotated reference images—were segmented by thresholding and quantified as the percentage of total peel area, with the whole-bunch silhouette isolated by background subtraction. For each time point, three bananas (n = 3) per packaging treatment were analyzed independently and mean ± SD values are reported. Browning values are expressed as the change in brown-area percentage relative to day 0.

Pulp-to-peel (P/P) ratio. Bananas were manually peeled; pulp and peel were weighed separately. Values are expressed as the percentage change in the pulp-to-peel mass ratio from day 0. An increase in P/P ratio reflects loss of peel mass relative to pulp, which occurs through two distinct routes that the ratio alone cannot separate: gradual peel dehydration during normal ripening, and when excessive, severe peel water loss that can itself compromise tissue integrity. A decrease in P/P ratio signals overripening-induced tissue collapse, wherein softened pulp adheres to the peel and disrupts clean separation. Because a rising P/P ratio is therefore not monotonically favorable, P/P values were interpreted jointly with the browning index and with peel/whole-fruit weight-loss data rather than in isolation.

Pulp and peel masses were determined immediately after manual separation using an analytical balance; weighing was completed within 2 min of peel detachment to minimize evaporative water loss.

Sugar content. Banana flesh (1.00 ± 0.01 g) was excised from the central portion of each finger, homogenized in 9 mL of deionized water using a handheld tissue homogenizer (BagMixer 400, Interscience Laboratory Inc., St. Nom, France) for 30 s at ambient temperature, and filtered through a 150-μm stainless-steel mesh. Soluble solid content was measured on a digital handheld refractometer (Atago Co., Ltd., Tokyo, Japan) with automatic temperature compensation, calibrated daily against deionized water at 20 °C. Each sample was measured in triplicate. Values are reported as Δ°Brix, defined as the change from day 0 after correction for the 10-fold dilution (i.e., multiplying the refractometer reading by 10) [34].

## 3. Results

### 3.1. Mechanical Properties of Active Films

Despite the 15 wt% mineral loading, the active films exhibited a tensile strength of 27.7 MPa (127% of the target value), elongation at break of 244% (102% of target), and tear strength of 990 N/cm (99% of target) (Table 2). These values indicate sufficient mechanical integrity for use as crate liner bags in industrial banana logistics.

All reported mechanical values represent the mean of five independent specimens (n = 5) and were determined according to the ASTM standards described in Section 2.3. The ‘target values’ reported in Table 2 correspond to the minimum specifications previously established for banana crate liner applications through industry-scale field trials.

### 3.2. Quality Changes Under Sealed Storage

#### 3.2.1. Browning Index

**Figure 1:**
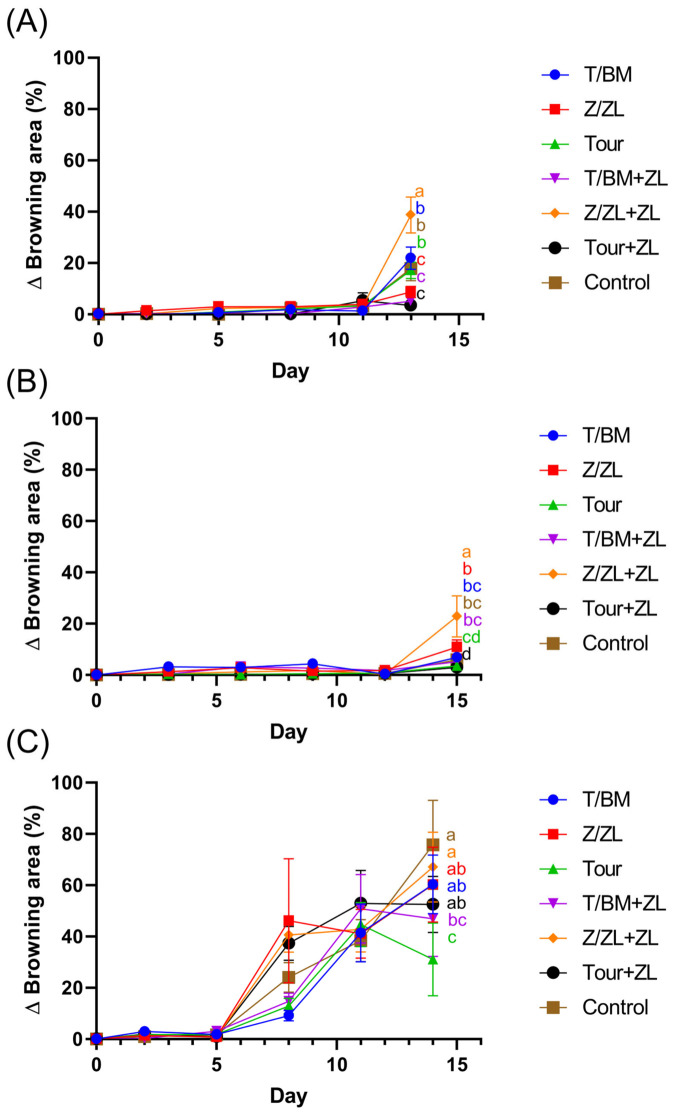
Changes in browning index of the bananas from (**A**) Ecuador, (**B**) Jeju, and (**C**) Mexico during sealed storage with different active packaging films. Different letters at the final point indicate significant differences (*p* < 0.05, Duncan’s multiple range test).

The visual progression of peel browning during sealed storage is documented in Figure S3. For Ecuador-origin bananas (Figure 1A), the browning index remained below 5% in most films until day 11. Minor negative values observed at early time points (e.g., Tour, day 2: 0.34%) were attributed to inter-specimen variability inherent in the destructive sampling protocol and interpreted as the absence of measurable browning. At day 13, pronounced differentiation among films appeared. Tour+ZL exhibited the lowest browning (3.48%), followed by T/BM+ZL (5.12%) and Z/ZL (8.71%). Tour (17.16%) and the control (17.88%) showed comparable and substantially higher browning, while T/BM (21.84%) exceeded the control. Z/ZL+ZL recorded the highest value at 38.70%.

For Jeju-origin bananas (Figure 1B), higher inter-specimen variability was observed overall. Most films maintained browning below 5% through day 12. At day 15, the lowest browning levels were observed in the Tour+ZL (2.99%) and Tour (3.54%) groups, while Z/ZL+ZL exhibited significantly higher browning at 22.86%. The top two films (Tour+ZL and T/BM+ZL) were consistent with those identified for Ecuador-origin bananas.

Mexico-origin bananas (Figure 1C) exhibited a qualitatively different pattern. Browning remained below 3% through day 5 for all films; however, by day 8, values surged to 9–46% across all treatments, with Z/ZL reaching 46.12%. By day 14, browning ranged from 31% (Tour) to 76% (control), confirming that no film effectively retarded browning in this origin.

#### 3.2.2. Pulp-to-Peel (P/P) Ratio

**Figure 2:**
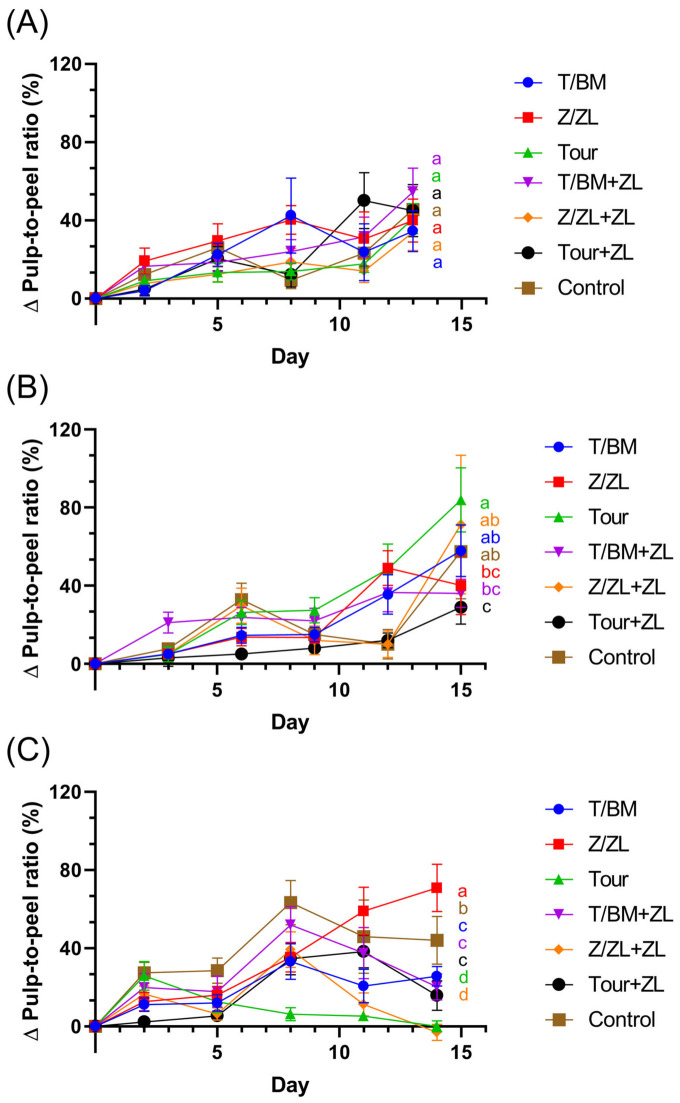
Changes in pulp-to-peel (P/P) ratio in bananas from (**A**) Ecuador, (**B**) Jeju, and (**C**) Mexico during sealed storage with different active packaging films. Different letters at the final point indicate significant differences (*p* < 0.05, Duncan’s multiple range test).

For Ecuador-origin bananas (Figure 2A), Tour and T/BM+ZL maintained monotonically increasing P/P ratios throughout storage, reaching terminal values of 40.94% for Tour and 54.55% for T/BM+ZL at day 13 (Figure 2A). This monotonic increase indicated the absence of tissue collapse. Tour+ZL remained relatively low during early storage (day 2–8: 4.86–12.20%) but showed an abrupt increase at day 11 (50.16%) followed by a slight decrease at day 13 (45.00%). In contrast, decreases in P/P ratio were observed for T/BM (day 8: 42.50% → day 11: 23.70%), Z/ZL (40.20% → 30.49%), control (day 5: 26.12% → day 8: 9.49%), and Z/ZL+ZL (18.73% → 14.10%), coinciding with films that exhibited higher browning.

For Jeju-origin bananas (Figure 2B), Tour+ZL exhibited the lowest terminal P/P ratio (28.99%) among all films, with a steady, monotonic increase from 0% to a terminal value of 28.99% without any decrease at any time point. Tour, in contrast, showed the highest terminal value (83.95%), indicating the most extensive peel dehydration despite its favorable browning performance. Z/ZL+ZL (day 6: 29.72% → day 9: 12.00% → day 12: 10.00%) and the control (32.80% → 15.00% → 10.00%) exhibited consecutive decreases over two intervals, consistent with overripening-associated tissue collapse.

For Mexico-origin bananas (Figure 2C), P/P ratios showed irregular fluctuation and progressive decreases for most films. Tour decreased continuously from 25.90% (day 2) to 0.04% (day 14); Z/ZL+ZL reached −3.08% on day 14. These patterns are consistent with the rapid browning observed in this origin.

#### 3.2.3. Change in Sugar Content

**Figure 3:**
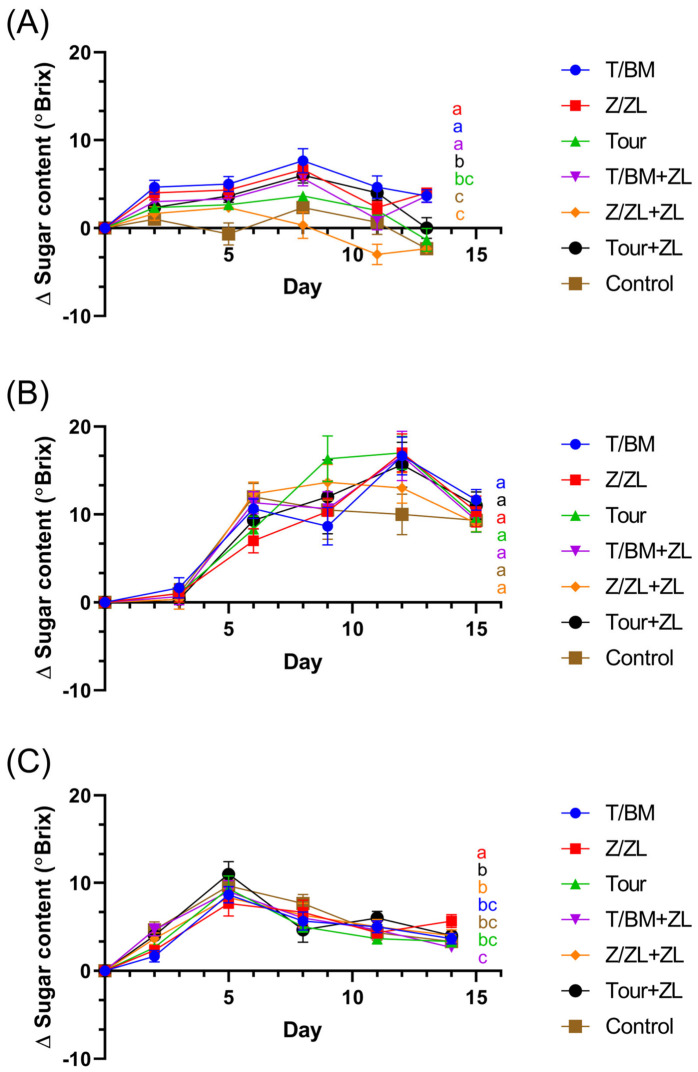
Changes in sugar content (°Brix) in bananas from (**A**) Ecuador, (**B**) Jeju, and (**C**) Mexico during sealed storage with different active packaging films. Different letters at the final point indicate significant differences (*p* < 0.05, Duncan’s multiple range test).

For Ecuador-origin bananas (Figure 3A), Tour exhibited the smallest Δ°Brix throughout storage (maximum: 3.66 at day 8), while Tour+ZL showed moderate changes (maximum: 6.00 at day 8). Z/ZL+ZL recorded negative values at days 11 (−3.00) and 13 (−2.33), consistent with overripening-associated sugar dilution from tissue water influx.

For Jeju-origin bananas (Figure 3B), most films reached peak Δ°Brix at day 12 (13.00 to 17.00) and declined by day 15. No statistically significant differences among films were detected at the final time point (Duncan’s test, *p* > 0.05).

For Mexico-origin bananas (Figure 3C), all films peaked at day 5 (7.67 to 11.00 Δ°Brix) and declined thereafter, with no significant inter-film differences, corroborating the absence of packaging effects observed for browning and P/P ratio.

### 3.3. Cross-Indicator Synthesis Under Sealed Storage

Across the three indicators, two consistent findings emerged for Ecuador and Jeju bananas. First, the two films containing 17% zeolite (Tour+ZL and T/BM+ZL) ranked among the lowest in browning at the terminal time point in both origins. Second, films for which P/P ratio decreased during storage (T/BM, Z/ZL+ZL, control) tended to exhibit higher browning, establishing the concurrent occurrence of P/P decline and elevated browning as a composite signature of overripening-induced tissue collapse.

For Mexico-origin bananas, browning surged 5–7 days earlier than in the Ecuador (day 13) or Jeju (day 15) bananas, and P/P ratio decreases were widespread. No film mitigated these changes in Mexico-origin bananas. Because fruit was sourced from retail outlets without documentation of postharvest handling, the failure cannot be attributed solely to intrinsic cultivar differences; differences in harvest maturity, cold-chain duration, or ethylene treatment history among supply chains are equally plausible explanations. These results collectively suggest that the efficacy of mineral-based active packaging depends critically on the physiological state of the fruit at the time of packaging, which under retail procurement conditions is confounded by origin-specific logistics that cannot be disentangled from intrinsic cultivar effects.

### 3.4. Quality Changes Under Perforated Storage

#### 3.4.1. P/P Ratio

**Figure 4:**
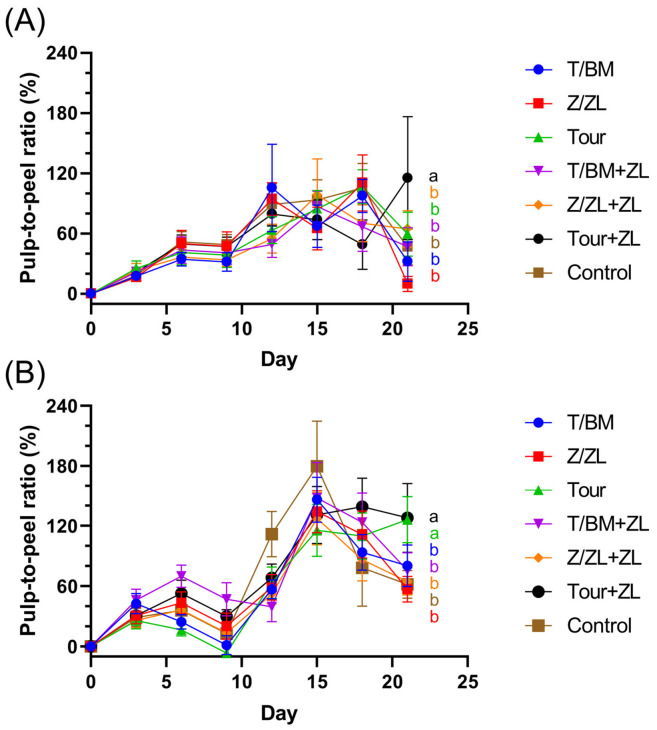
Changes in pulp-to-peel (P/P) ratio in bananas from (**A**) Ecuador and (**B**) Jeju during perforated storage with different active packaging films. Different letters at the final point indicate significant differences (*p* < 0.05, Duncan’s multiple range test).

Under perforated conditions, both bananas from Ecuador (Figure 4A) and Jeju (Figure 4B) exhibited substantially higher P/P ratios than under sealed conditions, reflecting accelerated peel dehydration due to ventilation. All films showed P/P values of 49–180% during days 12–18, with widespread decreases by day 21, indicating onset of overripening. The monitorable storage period was extended to 21 days (vs. 13–15 days sealed), but inter-film differences were attenuated.

#### 3.4.2. Sugar Content

**Figure 5:**
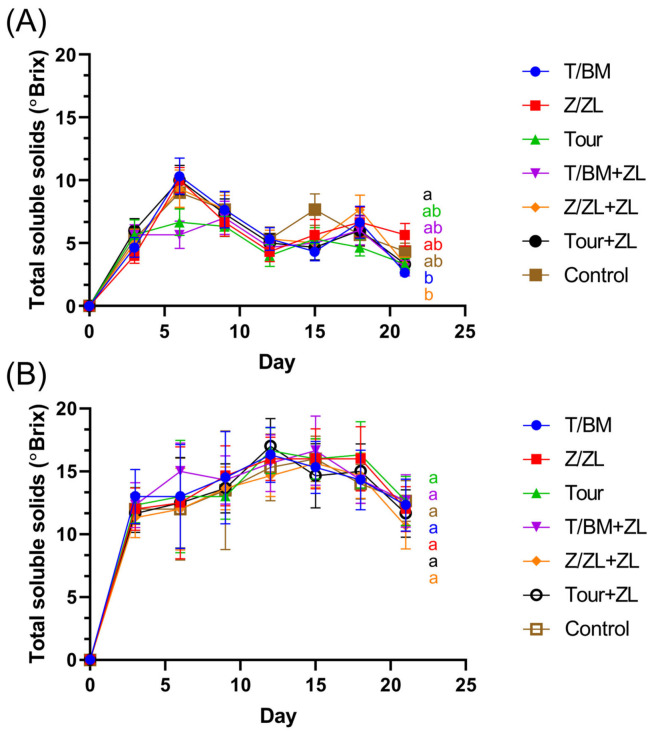
Changes in sugar content (°Brix) in bananas from (**A**) Ecuador and (**B**) Jeju during perforated storage with different active packaging films. Different letters at the final point indicate significant differences (*p* < 0.05, Duncan’s multiple range test).

Under perforated conditions, Ecuador bananas (Figure 5A) peaked in Δ°Brix at approximately day 6 and declined gradually; Jeju bananas (Figure 5B) peaked at approximately day 12. In both origins, inter-film differences at the final time point were smaller than those observed under sealed storage.

Perforated packaging extended the observation period by 6–8 days relative to sealed storage. However, the functional advantage of individual active films was more clearly expressed under sealed conditions, where restricted gas exchange likely maximized the adsorptive and catalytic functions of the mineral fillers.

## 4. Discussion

### 4.1. Role of Zeolite in Browning Suppression and Necessity of Composite Formulations

The most robust finding of this study is the reproducible ranking of zeolite-blended films (Tour+ZL and T/BM+ZL) as the lowest-browning formulations across two geographically distinct banana origins. The common feature of these two films was the inclusion of zeolite at 17 wt% alongside a secondary functional mineral (tourmaline or Ti/boehmite at 3 wt%). This reproducibility points to the microporous framework of zeolite and its capacity for ethylene physisorption as the principal mechanism underlying browning suppression [15,35].

Strikingly, the corresponding single-component counterparts—Tour and T/BM—showed markedly higher browning. In Ecuador-origin bananas, T/BM recorded 21.84% browning versus 17.88% for the unmodified control, indicating that Ti/boehmite alone did not confer browning suppression and may have marginally exacerbated it under the sealed storage conditions examined in this study. Several non-exclusive mechanisms may contribute to this outcome and warrant consideration. First, the γ-AlOOH framework of boehmite presents Lewis acid sites with lower ethylene binding affinity than the cation-exchange–active sites of zeolite, consistent with the generally reported advantage of zeolite-based sorbents over other aluminosilicates for olefin capture [21,23]. Second, Ti^4+^-loaded oxide surfaces are reported to initiate the partial oxidation of ethylene and related volatiles via hydroxyl radical generation in humid environments. This process yields aldehyde and peroxide intermediates that if not fully oxidized, could themselves promote peel oxidative browning [5,19]. Third, the pronounced hygroscopicity of boehmite may alter the internal humidity of the sealed pouch in a manner that modulates polyphenol oxidase (PPO) activity, which is recognized as the principal enzymatic driver of banana peel browning. Direct discrimination among these mechanisms will require headspace gas composition analysis (e.g., GC–MS) and film surface chemical characterization (XPS, FTIR), which were outside the scope of this screening study and are planned for follow-on work with the lead candidate formulations. Irrespective of the precise mechanism, these results demonstrate that composite formulations incorporating a high-capacity ethylene adsorbent (zeolite) are essential; single-mineral systems are insufficient regardless of their individual catalytic or radiative properties. Although headspace ethylene was not directly quantified in this screening study, the observed performance hierarchy provides indirect but internally consistent support for an ethylene-mediated mechanism: the two formulations containing 17 wt% zeolite (Tour+ZL and T/BM+ZL) reproducibly achieved the lowest browning across two independent banana origins, whereas single-mineral films lacking the high-capacity zeolite component did not.

### 4.2. P/P Ratio as a Dual Indicator: Peel Moisture Retention and Overripening Detection

The P/P ratio, interpreted as a measure of peel dehydration rate and tissue integrity, provided information complementary to the browning index. In Jeju bananas, Tour+ZL exhibited a low, monotonically increasing terminal P/P ratio (28.99%), whereas Tour reached the highest terminal value (83.95%) despite its low browning index (3.54%). Rather than indicating favorable performance, this divergence exposes a limitation of browning-only assessment: the low browning index of the Tour film did not reflect preserved tissue quality but coexisted with the most severe peel dehydration among all films. This is consistent with the established sequence in which excessive water loss compromises cell membrane integrity, decompartmentalizes polyphenol oxidase (PPO) and its substrates, and ultimately accelerates browning [8,35]. Under our 15-day observation window, the Tour film likely represented an early stage of this trajectory, in which dehydration was already advanced but had not yet manifested as brown pixel area. The addition of zeolite (Tour+ZL) moderated headspace humidity and suppressed this dehydration, yielding both low browning and a stable P/P ratio. The Tour case therefore does not contradict the water-loss/PPO/browning relationship. On the contrary, it illustrates precisely why a single browning metric is insufficient and why the complementary P/P (and weight-loss) indicators adopted here are necessary to distinguish genuine quality retention from a transient, dehydration-driven suppression of visible browning.

A decrease in P/P ratio was observed for T/BM, Z/ZL+ZL, and the control in both Ecuador and Jeju bananas, and these same films exhibited higher browning. The coincidence of P/P decline with elevated browning across multiple films and origins supports the utility of P/P decrease as a diagnostic marker of overripening-associated tissue collapse. This cross-validated approach—using browning as the primary quality indicator and P/P decline as a confirmatory signal of overripening—may reduce the risk of misinterpretation inherent in relying on a single metric.

### 4.3. Origin-Dependent Efficacy and Implications for Industrial Application

The failure of all films to retard browning in Mexico-origin bananas, where rapid browning onset (day 8) preceded that of Ecuador (day 13) and Jeju (day 15) bananas by 5–7 days, constitutes a practically significant finding. The simultaneous occurrence of rapid browning and progressive P/P decline in Mexico bananas points to a climacteric surge that exceeded the ethylene-sorption capacity of every film tested. Because all bananas were purchased from retail outlets without control of postharvest handling history, the observed origin effect may reflect differences in cold-chain management during transport, maturity stage at purchase, or intrinsic origin physiology [7,29,36,37]. Resolving these confounding factors would require future experiments with bananas harvested from known plantations and shipped under controlled conditions.

From an industrial perspective, these results imply that active packaging cannot be deployed as a universal solution; rather, its adoption must be matched to the physiological state and origin characteristics of the target fruit. Preliminary screening such as the present work is therefore a necessary step before field-scale implementation.

### 4.4. Limitations and Future Directions

Several limitations constrain the interpretation of the present results. First, the destructive nature of the measurements necessitated the use of a different banana specimen at each time point, so inter-time-point variability includes both packaging effects and specimen-to-specimen variation. Moreover, because the fruit was procured from retail outlets, each lot had passed through multiple, undocumented handling and distribution touchpoints before purchase; this batch-level heterogeneity, together with the limited replication (n = 3) inherent to the destructive sampling protocol, may affect the reproducibility of the absolute values reported here and should be addressed in future work through larger sample sizes and fruit of documented provenance. Second, headspace ethylene concentration was not monitored, precluding direct quantification of the relationship between ethylene removal and quality retention. Third, the bananas were sourced from commercial retail markets without documented postharvest histories; in particular, the commercial ethylene-ripening status of each lot at purchase could not be verified. Because origin, cultivar, cold-chain duration, and ripening-induction history are inherently confounded under retail procurement, the origin-dependent efficacy reported here should be interpreted as a combined effect of these factors rather than a pure cultivar effect—consistent with our framing of the study as a preliminary screening and with the controlled, known-provenance trials proposed below [38].

To address these limitations and extend the present findings, future work should: (a) verify the efficacy of Tour+ZL and T/BM+ZL under temperature-controlled conditions with increased replication (n ≥ 5); (b) incorporate real-time ethylene monitoring by gas chromatography or electrochemical sensors; (c) elucidate the mechanism by which tourmaline modulates peel browning without preventing moisture loss; and (d) compare bananas of uniform cultivar background under defined postharvest handling regimes to decouple origin effects from logistics effects.

## 5. Conclusions

Six mineral-loaded LDPE active packaging films were screened for postharvest quality retention of bananas from three origins. The films retained adequate mechanical properties at 15 wt% mineral loading (tensile strength: 27.7 MPa; elongation: 244%).

Under sealed storage, the two zeolite-blended formulations (Tour+ZL and T/BM+ZL) consistently exhibited the lowest browning across the Ecuador- and Jeju-origin bananas, with Tour+ZL recording the lowest values overall (Ecuador: 3.48%; Jeju: 2.99%). Tour+ZL also demonstrated the lowest terminal P/P ratio (28.99%) in Jeju bananas, with no decrease throughout storage, indicating superior peel moisture retention and tissue integrity. In contrast, the single-component T/BM film showed browning equal to or exceeding that of the unmodified control, confirming that zeolite incorporation is essential for effective performance.

Concurrent decreases in P/P ratio and elevated browning were observed for T/BM, Z/ZL+ZL, and the control, supporting the use of P/P decline as a confirmatory indicator of overripening-induced tissue collapse. Mexico-origin bananas showed no packaging effect under any film, highlighting strong origin-dependence of active packaging efficacy.

This screening study identified Tour+ZL as the priority candidate for subsequent controlled validation trials and proposes a browning–P/P cross-verification framework for postharvest quality assessment.

## Figures and Tables

**Table 1:** Composition of active packaging films used in this study.

Designation	Functional Filler Composition	Total Mineral Loading in M/B
T/BM	Ti/boehmite (20%)	15%
Z/ZL	Zn/zeolite (20%)	
Tour	Tourmaline (20%)	
T/BM+ZL	Ti/boehmite (3%) + Zn/zeolite (17%)	
Z/ZL+ZL	Zn/zeolite (3%) + zeolite (17%)	
Tour+ZL	Tourmaline (3%) + Zn/zeolite (17%)	
Control	Neat PE (no filler)	0%

**Table 2:** Mechanical properties of active packaging films.

Parameters	Target Value	Measured Value	Achievement Rate (%)
Tensile strength (MPa)	22	27.7 ± 2.1	127
Elongation at break (%)	240	244 ± 78	102
Tear strength (N/cm)	1000	989 ± 41	99

## Data Availability

The original contributions presented in this study are included in the article/Supplementary Materials. Further inquiries can be directed to the corresponding author.

## Supplementary Materials

The following supporting information can be downloaded at: https://www.mdpi.com/article/10.3390/foods15111989/s1. Figure S1. Appearance of the packaging films used in this study. (A) Active packaging films produced with different mineral fillers; arrows indicate, from left to right, T/BM, Z/ZL, Tour, T/BM+ZL, Z/ZL+ZL, Tour+ZL, and the neat-PE control. (B) Appearance of the perforated pouch (ø ≈ 2 mm perforations) used in the Phase 2 (perforated storage) trials. Figure S2. Representative images of bananas of the three origins sealed in standardized pouches (25 cm × 19 cm) at the start of storage (day 0) under Phase 1 (sealed storage) conditions: (A) Ecuador, (B) Jeju, and (C) Mexico. Figure S3. Visual progression of peel and pulp appearance of bananas of the three origins during Phase 1 (sealed storage): (A) Ecuador, (B) Jeju, and (C) Mexico. For each origin, images are shown at the origin-specific sampling intervals (Ecuador: days 0, 2, 5, 8, 11, 13; Jeju: days 0, 3, 6, 9, 12, 15; Mexico: days 0, 2, 5, 8, 11, 14; left to right), with the peel and the separated pulp shown for each time point.

## Abbreviations

The following abbreviations are used in this manuscript:

LDPE	Low-density polyethylene
HDPE	High-density polyethylene
PE	Polyethylene
PPO	Polyphenol oxidase
P/P ratio	Pulp-to-peel ratio
Δ°Brix	Change in degrees Brix (sugar content)
T/BM	Ti^4+^/boehmite-loaded film
Z/ZL	Zn^2+^/zeolite-loaded film
Tour	Tourmaline-only film
ZL	Zeolite (used as suffix indicating 17 wt% zeolite addition)
T/BM+ZL	Ti^4+^/boehmite (3 wt%) + zeolite (17 wt%) film
Z/ZL+ZL	Zn^2+^/zeolite (3 wt%) + zeolite (17 wt%) film
Tour+ZL	Tourmaline (3 wt%) + zeolite (17 wt%) film
