# Supplementary figures and images for: Preliminary Screening of Mineral-Based Active Packaging Films for Banana Postharvest Quality: Origin-Dependent Efficacy and Superiority of Tourmaline-Based Formulations
Source: Foods. 2026 Jun 3;15(11):1989. doi: 10.3390/foods15111989 (PMC13256096; doi:10.3390/foods15111989)

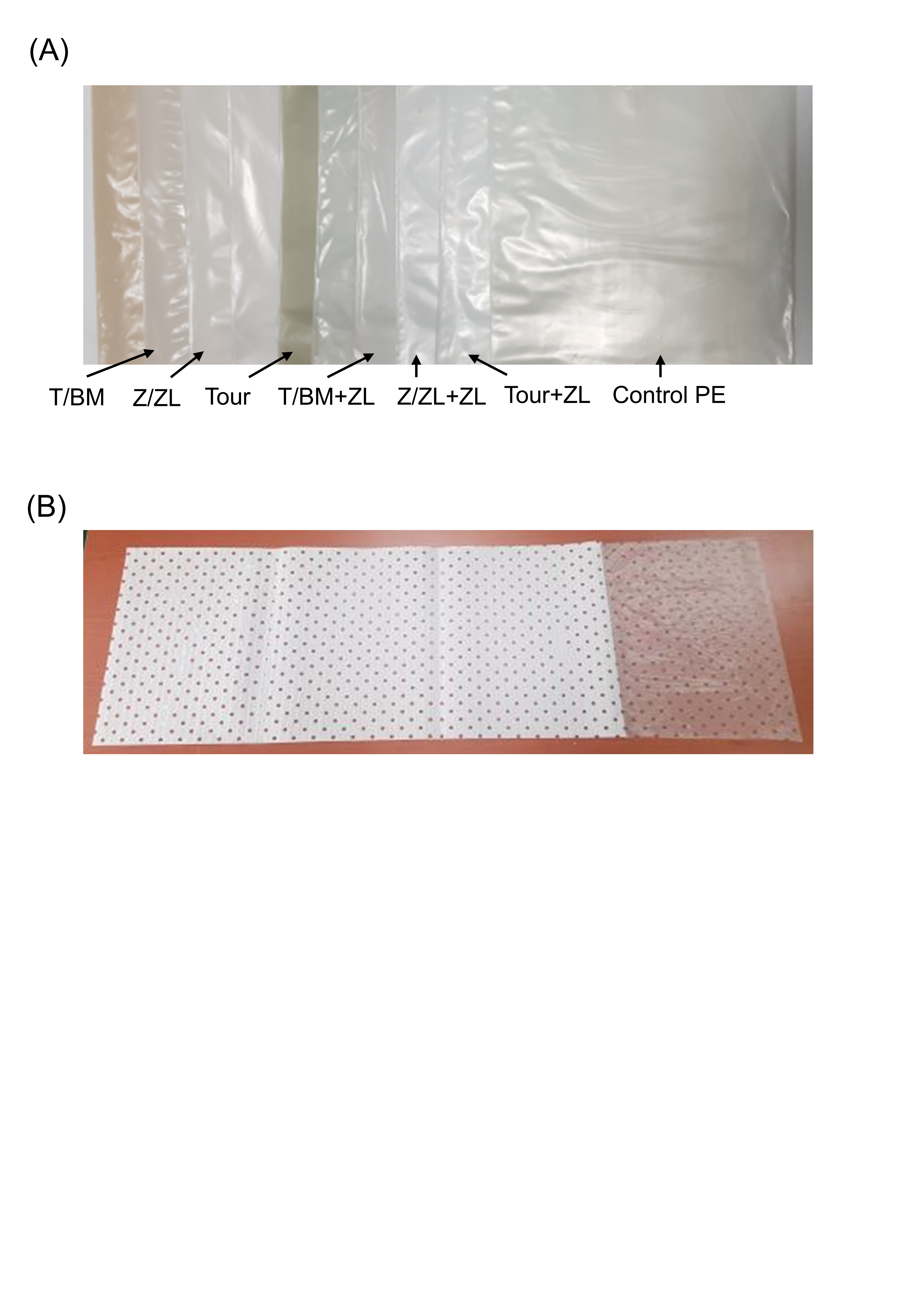

Supplement: Supplementary file 1 [file foods-15-01989-s001.zip › Figure. S1.tif]

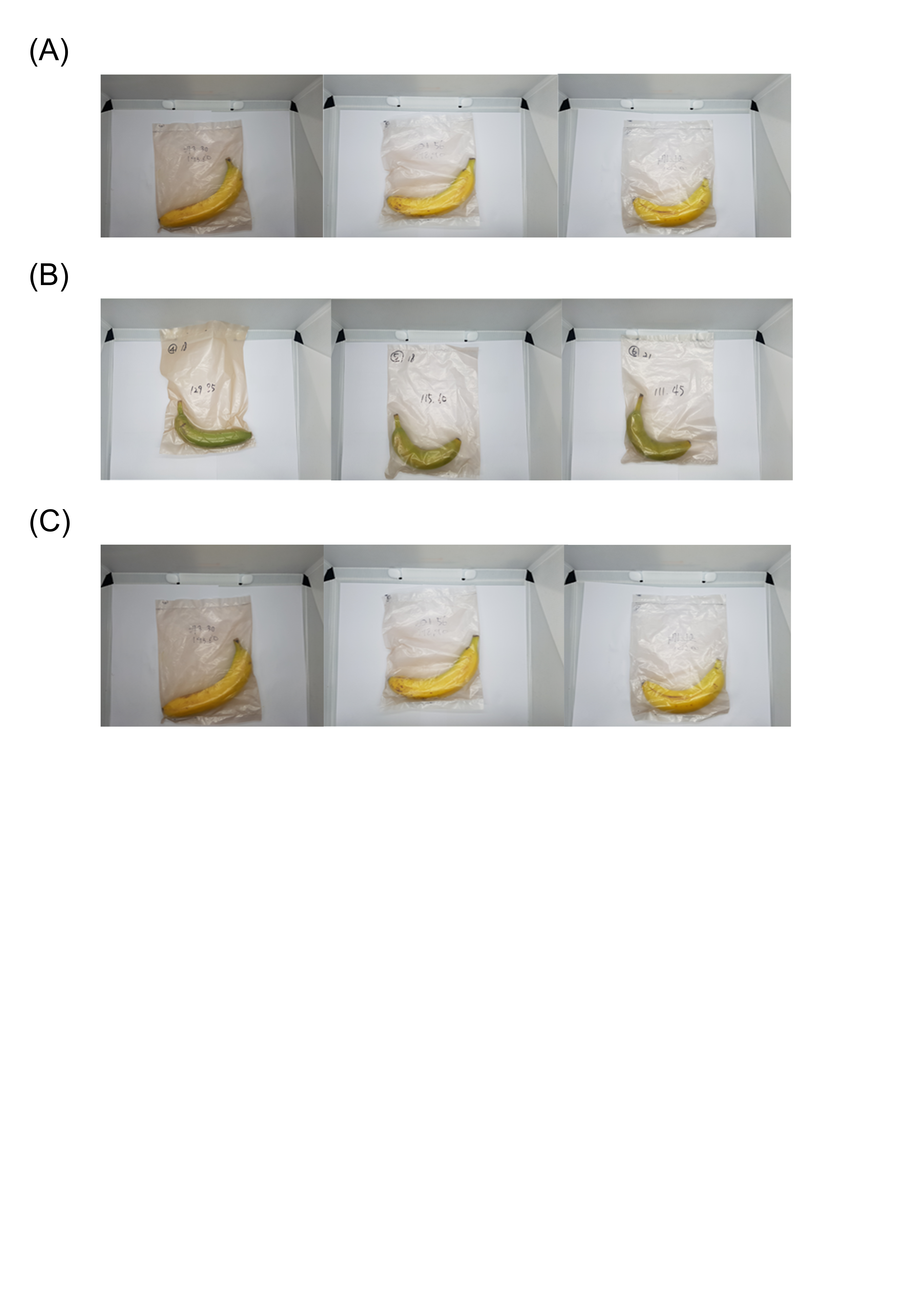

Supplement: Supplementary file 1 [file foods-15-01989-s001.zip › Figure. S2.tif]

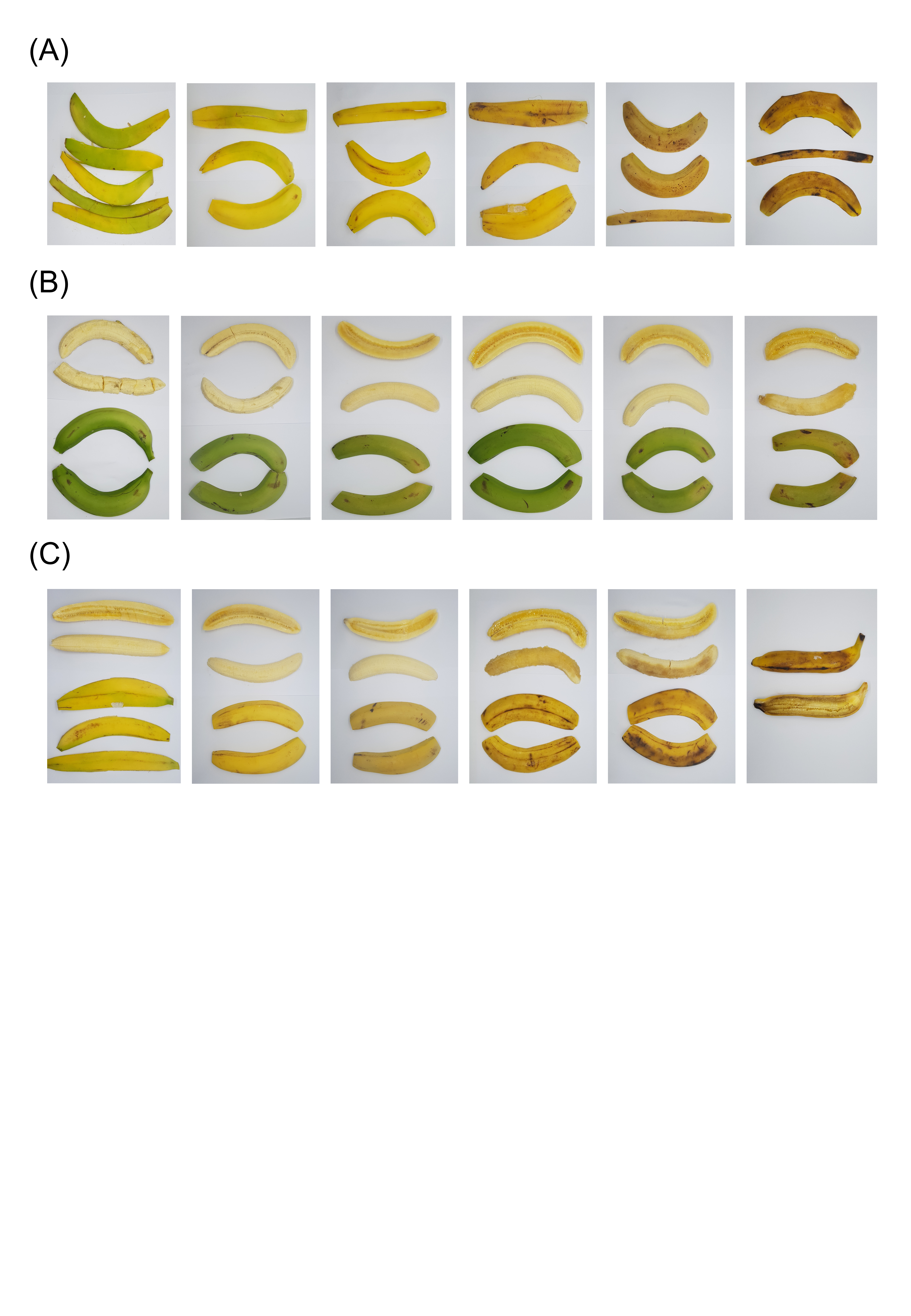

Supplement: Supplementary file 1 [file foods-15-01989-s001.zip › Figure. S3.tif]
